# A Machine Learning Approach for Hot-Spot Detection at Protein-Protein Interfaces

**DOI:** 10.3390/ijms17081215

**Published:** 2016-07-27

**Authors:** Rita Melo, Robert Fieldhouse, André Melo, João D. G. Correia, Maria Natália D. S. Cordeiro, Zeynep H. Gümüş, Joaquim Costa, Alexandre M. J. J. Bonvin, Irina S. Moreira

**Affiliations:** 1 Centro de Ciências e Tecnologias Nucleares, Instituto Superior Técnico, Universidade de Lisboa, Estrada Nacional 10 (ao km 139,7), 2695-066 Bobadela LRS, Portugal; ritamelo@ctn.ist.utl.pt (R.M.); jgalamba@ctn.tecnico.ulisboa.pt (J.D.G.C.); 2 CNC—Center for Neuroscience and Cell Biology; Rua Larga, Faculdade de Medicina, Polo I, 1ºandar, Universidade de Coimbra, 3004-504 Coimbra, Portugal; 3 Department of Genetics and Genomics and Icahn Institute for Genomics and Multiscale Biology, Icahn School of Medicine at Mount Sinai, New York, NY 10029, USA; robert.fieldhouse@mssm.edu (R.F.); zeynep.gumus@gmail.com (Z.H.G.); 4 REQUIMTE (Rede de Química e Tecnologia), Faculdade de Ciências da Universidade do Porto, Departamento de Química e Bioquímica, Rua do Campo Alegre, 4169-007 Porto, Portugal; asmelo@fc.up.pt (A.M.); ncordeir@fc.up.pt (M.N.D.S.C.); 5 CMUP/FCUP, Centro de Matemática da Universidade do Porto, Faculdade de Ciências, Rua do Campo Alegre, 4169-007 Porto, Portugal; Jpcosta@fc.up.pt; 6 Bijvoet Center for Biomolecular Research, Faculty of Science—Chemistry, Utrecht University, Utrecht 3584CH, The Netherlands; a.m.j.j.bonvin@uu.nl

**Keywords:** protein-protein interfaces, hot-spots, machine learning, Solvent Accessible Surface Area (SASA), evolutionary sequence conservation

## Abstract

Understanding protein-protein interactions is a key challenge in biochemistry. In this work, we describe a more accurate methodology to predict Hot-Spots (HS) in protein-protein interfaces from their native complex structure compared to previous published Machine Learning (ML) techniques. Our model is trained on a large number of complexes and on a significantly larger number of different structural- and evolutionary sequence-based features. In particular, we added interface size, type of interaction between residues at the interface of the complex, number of different types of residues at the interface and the Position-Specific Scoring Matrix (PSSM), for a total of 79 features. We used twenty-seven algorithms from a simple linear-based function to support-vector machine models with different cost functions. The best model was achieved by the use of the conditional inference random forest (c-forest) algorithm with a dataset pre-processed by the normalization of features and with up-sampling of the minor class. The method has an overall accuracy of 0.80, an *F1*-score of 0.73, a sensitivity of 0.76 and a specificity of 0.82 for the independent test set.

## 1. Introduction

Among all of the cellular components of living systems, proteins are the most abundant and the most functionally versatile. The specific interactions formed by these macromolecules are vital in a wide-range of biological pathways [1]. Protein-protein interactions involved in both transient and long-lasting networks of specific complexes play important roles in many biological processes [2,3,4]. Characterizing the critical residues involved in these interactions by both experimental and computational methods is therefore crucial to a proper understanding of living systems. Furthermore, only by gaining a complete understanding at atomistic detail can new methods be developed to modulate their binding [5,6].

Protein-protein interfaces often involve a large number of residues. However, it is generally recognized that small regions of a few residues, termed “Hot-Spots (HS)”, are essential for maintaining the integrity of the interface. The development of techniques to identify and characterize protein-based interfaces has become widespread. Experimental Alanine Scanning Mutagenesis (ASM) continues to be a valuable technique for both detecting and analyzing protein-binding interfaces. The contribution of a residue to the binding energy is measured by the binding free energy difference (ΔΔ*G*_binding_) between the wild-type (WT) and mutant complex upon mutation of a specific residue to alanine [7]. Bogan and Thorn [8] defined the residues with ΔΔ*G*_binding_ ≥ 2.0 kcal·mol^−1^ as HS; and the residues with ΔΔ*G*_binding_ < 2.0 kcal·mol^−1^ as Null-Spots (NS). Experimental methods for identifying HS are based on molecular biology techniques that are accurate, but still complex, time-consuming and expensive [9]. Highly efficient computational methods for predicting HS can provide a viable alternative to experiments. Molecular Dynamics (MD) simulations can be used to predict changes in the binding strength of protein complexes by calculating the free energy difference from an initial to a final state [10,11]. However, due to the complexity and typical large size of protein-protein complexes, these methods are still computationally expensive. Recently, machine learning approaches trained on various features of experimentally-determined HS residues have been developed in order to predict HS in new protein complexes [6,12,13,14].

In previous work, we have investigated feature-based methods combining Solvent Accessible Surface Area (SASA) descriptors calculated from static structures and MD ensembles and trained predictors using a Support Vector Machine (SVM) algorithm [15]. However, we only applied these to a small number of complexes, and the prediction performance was hampered by a high number of false positives. More recently, we added an extra feature (residue evolutionary sequence conservation) on a significantly larger dataset. In that study, we explored additional Machine Learning (ML) techniques, which led us to develop a more accurate and time-efficient HS detection methodology. This resulted in new HS predictor models for both protein-protein and protein-nucleic acid interactions, and we implemented the best performing models into two web tools [14].

In this study, we significantly expand both the number of studied protein-protein complexes and the number of 3D complex structure-based features used for prediction, including: interface size, the type of interaction between residues at the interface of the complex and the number of different types of residues at the interface. To the evolutionary sequence-based features, we added the Position-Specific Scoring Matrix (PSSM), for a total of 79 features. We have further tested a total of 27 algorithms from a simple linear-based function to support-vector machine models with different cost functions. The best predictor, based on a conditional inference random forest (c-forest) algorithm, achieves an overall performance characterized with an *F*1-score of 0.73, an accuracy of 0.80, a sensitivity of 0.76 and a specificity of 0.82. To the best of our knowledge, these values are higher than all other available prediction techniques.

## 2. Results

In the current study, we have used the Classification And Regression Training (Caret) Package [16] from the R software [17], which provides a unified interface with a large number of built-in classifiers, in order to train an HS predictor. The dataset used for this purpose includes 545 amino acids from 53 complexes (140 HS and 405 NS). We calculated the percentage of the different types of amino acids within the NS set (Ser: 7.4; Gly: 1.5; Pro: 2.0; Val: 3.2; Leu: 2.7; Ile: 5.2; Met: 1.0; Cys: 0.7; Phe: 4.7; Tyr: 5.9; Trp: 4.9; His: 4.4; Lys 8.9; Arg: 10.6; Gln: 5.4; Asn: 6.2; Glu: 9.9; Asp: 7.2; Thr: 8.1) and within the HS set (Ser: 2.1; Gly: 2.9; Pro: 2.9; Val: 3.6; Leu: 7.1; Ile: 4.3; Met: 0.0; Cys: 0.0; Phe: 6.4; Tyr: 20.0; Trp: 5.7; His: 2.1; Lys 7.1; Arg: 6.4; Gln: 2.1; Asn: 5.0; Glu: 7.1; Asp: 10.7; Thr: 4.3). For both sets, there is a natural expected tendency for a higher percentage of large hydrophobic or charged residues at the interfaces, in particular Tyr. Although different patterns could influence the training of a robust classifier, we have previously successfully constructed models that were bias-free for all different amino acids [14]. We randomly split this dataset (see for details Supplementary Information Table S1) into a training set consisting of 70% of data (382 mutations) and an independent test set (163 mutations, 30%). This is a standard division scheme demonstrated to give a good result. All 27 classification models (listed in the Methods Section) were tested using 10-fold cross-validation repeated 10 times in order to avoid overfitting and to obtain the model’s generalization error. This means that the training set was split randomly into ten isolated parts, using nine of the ten parts to train the model and taking the remaining fold of data to test the final performance of the model. This process was repeated ten times. The performance of the five best algorithms for each tested condition was independently evaluated on the test set to ensure an unbiased assessment of the accuracy of the final model.

The 79 features used in this work have different scales (i.e., the range of the raw data varies significantly), and therefore, we have performed feature normalization or data standardization of the predictor variables at the training set by centering the data, i.e., subtracting the mean and normalizing it by dividing by the standard deviation. The same protocol was followed for the test set taking into account the use of the training mean and standard deviation to ensure a good estimation of the model quality and generalization power. As we have a high-dimensional dataset (79 features), we have also applied Principal Components Analysis (PCA) to reduce the dimensionality of the data. PCA works by establishing an orthogonal transformation of the data to convert a set of possible correlated variables into a set of linearly-uncorrelated ones, the so-called principal components.

**Figure 1:**
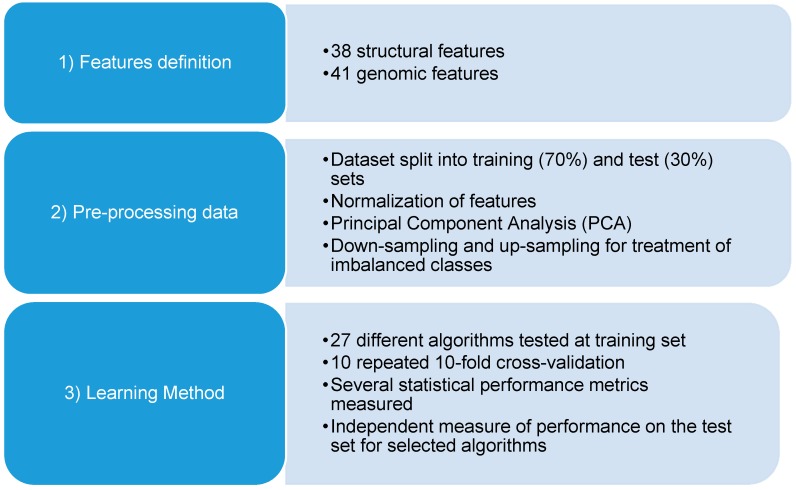
The flowchart of the current work.

One of the main concerns when applying classification to the detection of HS is the natural imbalance of the data. As expected, the number of HS is lower than the number of NS at a protein-protein interface, as indicated by the presence of 185 HS and 360 NS in the main dataset. In ML classification methods, the disparity of the frequencies of the observed classes may have a very negative impact on the models’ performance. To overcome this problem, we have tried two different subsampling techniques for the training set: down-sampling and up-sampling. In the first, there is a random sub-setting of all classes at the training set with their class frequency matching the least prevalence class (HS), whereas in the up-sampling, the opposite is happening with random sampling (with the replacement) of the minority class (HS) to reach the same size as the majority class (NS). Different conditions were thus established: (i) Scaled; (ii) Scaled Up; (iii) Scaled Down; (iv) PCA; (v) PCA Down; and (vi) PCA Up. Various statistical metrics (described in detail in the Methods Section) were adopted to evaluate the performance of the algorithms tested: Area Under the Receiver Operator Curve (AUROC), accuracy, True Positive Rate (TPR), True Negative Rate (TNR), Positive Predictive Value (PPV), False Positive Rate (FPR), False Negative Rate (FNR) and *F*1-score. Figure 1 illustrates the workflow followed in this study.

The results for the training set for the best five algorithms for each of the six conditions studied are listed in Table 1. All statistical metrics obtained for the complete set of algorithms can be found in Supplementary Information Table S2, in which a more straightforward comparison by type of method can be made. The best classifiers seem to be almost constant in all six different pre-processing conditions, including one neuronal network (avNNET: model averaged Neural Network) and two tree-based methods (C5.0 Tree, C5.0 Rules). The fourth and fifth classifiers vary from nnet (neuronal network), to c-forest, GBM (stochastic gradient boosting machine) and svmRadialSigma (support vector machines with the Radial basis function kernel). The up-sampling of the HS class seems to improve the classifier performance presenting AUROC values higher than 0.80 in the majority of the cases.

The performance of a classifier on the training set from which it was constructed gives a poor estimate of its accuracy in new cases. Furthermore, overfitting on algorithms without regularization terms (such as decision trees and neural networks) is harder to address on the training set. Therefore, the true predictive accuracy of the classifier was estimated on a separate test set corresponding to 30% of the main dataset. Table 2 summarizes the performance on the independent test set for the best classifiers shown in Table 1.

**Figure 2:**
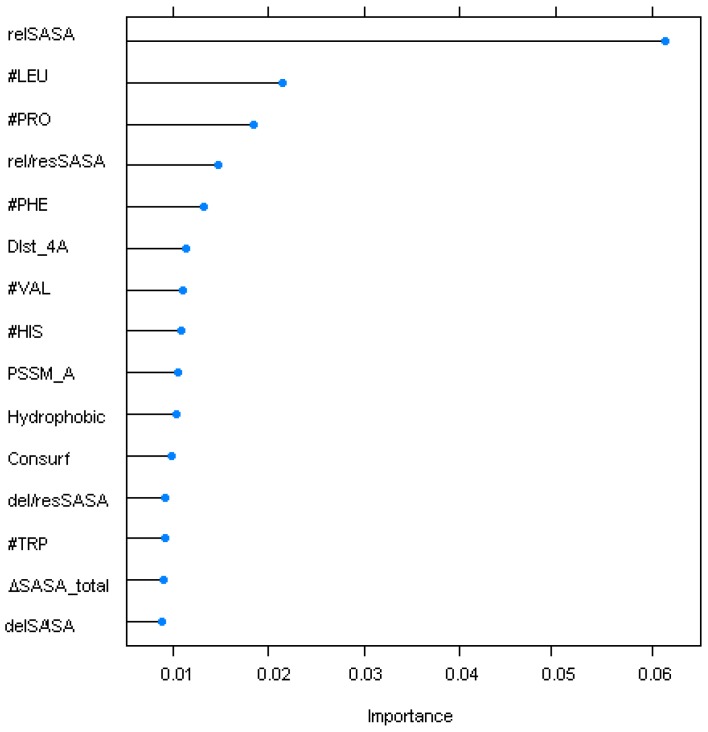
Top 15 variables for the c-forest method. SASA, Solvent Accessible Surface Area; #, Number of residues

From all of methods, c-forest, trained on the normalized up-scaling set, had the highest performance metrics on both training and test sets. It was therefore chosen as a final model. In our analysis of this classifier (Figure 2), we observed that the key features are structural ones: specifically, relSASA_i_, ΔSASA_i_, the number of contacts established by the interfacial residues at 4 Å and the number of LEU, VAL and HIS residues at the interface. All of these features were calculated using built-in functions of the VMD package [18] and in-house scripts.

To validate the accuracy of the best predictor, we performed the HS predictions with other methods reported in the literature, such as Robetta [19], KFC2-A (Knowledge-based FADE and Contacts) [20], KFC2-B [20] and CPORT (Consensus Prediction Of interface Residues in Transient complexes)(not specialized in HS prediction, but instead, a protein-protein interface predictor) [21] on the same training and test sets. The comparison among these ML methods (Table 3) demonstrates that our new method achieves the best performance with *F*1-scores/AUROC values of 0.73/0.78 on the test set against 0.39/0.62, 0.56/0.66, 0.42/0.67 and 0.43/0.54 for Robetta, KFC2-A, KFC2-B and CPORT, respectively.

## 3. Discussion

Machine learning is an area of artificial intelligence that is data driven with a focus on the development of computational techniques for making inferences or predictions. It has become widely used in a variety of areas due to its reduced application time and high performance. Over the past few years, a few algorithms have been applied for the specific problem in this study: the detection of hot-spots at protein-protein interfaces [13,14,15,22,23,24,25,26,27,28,29,30,31,32,33,34,35].

Here, neural networks and tree-based methods were highlighted as some of the high performance classifiers. Neural networks are inspired by biological nervous systems transmitting the information by a vast network of interconnecting processing elements (neurons). Decision trees organize the knowledge extracted from a hierarchy by using simple tests over the features of the training set. Both have been shown in the past to be promising ML algorithms in the bioinformatics field. Random forests were also shown to be able to predict the impact of each variable in high dimensional problems even in the presence of complex interactions [36]. In particular, c-forest [36], an implementation of the random forest and bagging ensemble method that uses conditional inference trees as base learners, achieved the top performance (Table 2) with a high F1-score of 0.93 on the training set using a 10 repeated 10-fold cross-validation. The values in the independent test (F1 score 0f 0.73) were also very high compared to the ones currently reported in the literature and surpassing all of the other methods tested in this study (Table 3; SBHD (Sasa-Based Hot-spot Detection) 0.61, Robetta 0.39, KFC2-A 0.56, KFC2-B 0.42 and CPORT 0.42). One important aspect that seemed to improve the results compared to our previous approaches (SBHD) was the use of in-built R techniques to balance the training data: up-scaling of the data led to a substantial improvement of the F1-score and to a decrease of the FPR to about 0.19 on the independent test set. In this particular classifier, the first seven features with higher importance were all structure-based: two already used in previous versions of our algorithm (ΔSASA_i_ and relSASA_i_, check Material and Methods) and five new ones (the number of residues at a 4 Å distance and the number of LEU, VAL, HIS and PRO residues at the interface). The PSSM value for the TYR residues, one of the most common residues as HS, was the first genomic-based feature to be ranked as important.

## 4. Material and Methods

### 4.1. Dataset Construction

We constructed a database of complexes by combining information from the Alanine Scanning Energetics database (ASEdb) [37], the Binding Interface Database (BID) [38] and the SKEMPI (Structural database of Kinetics and Energetics of Mutant Protein Interactions) [39] and PINT (Protein-protein Interactions Thermodynamic Database) [40] databases, which provide both experimental ΔΔ*G*_binding_ values for interfacial residues and tridimensional (3D) X-ray structure information. The protein sequences were filtered to ensure a maximum of 35% sequence identity for at least one protein in each interface. Crystal structures were retrieved from the Protein Data Bank (PDB) [41], and all water molecules, ions and other small ligands were removed. Our final dataset consists of 545 mutations from 53 different complexes.

### 4.2. Sequence/Structural Features

From a structural point of view, we compiled 12 previously-used different SASA descriptors for all interfacial residues [14,15]: (i) _comp_SASA_i_, the solvent accessible surface area of residue *i* in the complex form; (ii) _mon_SASA_i_, the residue SASA in the monomer form; (iii) ΔSASA_i_, the SASA difference upon complexation (Equation (1)); (iv) relSASA_i_, the ratio between ΔSASA for each residue and the _mon_SASA_i_ value for the same residue (Equation (2)). A further four features (_comp/res_SASA_i_, _mon/res_SASA_i_, _Δ/res_SASA_i_ and _rel/res_SASA_i_), defined by Equations (3)–(6), were determined applying amino acid standardization by dividing the previous features by the average protein _res_SASA_r_ values as determined by Miller and colleagues [42,43], with r being the respective residue type. Four additional, amino-acid standardized features were calculated by replacing the values determined by Miller by our own protein averages _ave_SASA_r_ for each amino acid type in its respective protein: _comp/ave_SASA_i_, _mon/ave_SASA_i_, _Δ/ave_SASA_i_ and _rel/ave_SASA_i_, defined in Equations (7)–(10).

(1) ΔSASAi=|SASAicomp−SASAimon|

(2) SASAirel=ΔSASAiSASAimon

(3) SASAicomp/res=SASAicompSASArres

(4) SASAimon/res=SASAimonSASArres

(5) SASAiΔ/res=ΔSASAiSASArres

(6) SASAirel/res=relSASAiSASArres

(7) SASAicomp/ave=SASAicompSASArave

(8) SASAimon/ave=SASAimonSASArave

(9) SASAiΔ/ave=ΔSASAiSASArave

(10) SASAirel/ave=relSASAiSASArave

As the SASA features described in Equations (3)–(10) are rather small, the results presented here were multiplied by a factor of 10^3^.

We further introduced two features directly related to the size of the interface: the total number of interfacial residues and the ΔSASA_total_ (sum of the ΔSASA_i_ of all residues at the protein-protein binding interfaces). Twenty other features were added by splitting the total number of interface residues into the 20 amino acid types. Four contact features were also calculated: (i) the number of protein-protein contacts within 2.5 Å and (ii) 4.0 Å distance cut-offs, respectively; (iii) the number of intermolecular hydrogen bonds; and (iv) the number of intermolecular hydrophobic interactions. In-house scripts using the VMD molecular package [18] were used for all of these calculations. We used in total 38 structural features in our study.

To utilize evolutionary sequence conservation information, we used the ConSurf server [44] that calculates a conservation score for each amino acid at an interfacial position for a complex, based on known sequences in different organisms. We also computed, PSSM using BLAST [45,46], as well as the weighted observed percentages, introducing them as 40 new features for all interfacial residues. Positive values in this matrix appear for substitutions more frequent than expected by random chance, and negative values indicate that the substitution is not frequent. Therefore, a total of 41 evolutionary sequence-related features were added to the structural features, resulting in 79 features in total for this study.

### 4.3. Machine Learning Techniques

We first pre-processed the dataset by eliminating missing values or NZV (Near Zero Variance) features. Next, as mentioned in the Results section, we normalized the dataset and performed PCA. The algorithms tested were: avNNet (model averaged Neural Network); bagEarth (bagged MARS (multivariate adaptive regression splines)); bagEarthGCV Bagged MARS using gCV pruning; bagFDA (bagged Flexible Discriminant Analysis); C5.0Rules (single C5.0 Ruleset); C5.0Tree (single C5.0 Tree); c-forest (conditional inference random forest); ctree (conditional inference tree); ctree2 (conditional inference tree); earth (multivariate adaptive regression spline); fda (flexible discriminant analysis); gaussprLinear (Gaussian process); GBM (stochastic gradient boosting machine); gcvEarth (multivariate adaptive regression splines); hdda (high dimensional discriminant analysis); knn (k-nearest neighbors); lda (linear discriminant analysis); lda2 (linear discriminant analysis); multinom (penalized multinomial regression); nnet (neuronal networks); nb (naive Bayes); pda2 (penalized discriminant analysis); svmLinear (Support Vector Machines with Linear Kernel); svmLinear2 (Support Vector Machines with Linear Kernel); svmPoly (Support Vector Machines with Polynomial Kernel); svmRadial (support vector machines with the Radial basis function kernel); svmRadialCost (support vector machines with the Radial basis function kernel); svmRadialSigma (support vector machines with the Radial basis function kernel); svmRadialWeights (support vector machines with class Weights).

The validity and performance of the various methods was determined by measuring the Area Under the Receiver Operator Curve (AUROC), the accuracy (Equation (11)), True Positive Rate (TPR/recall/sensitivity, Equation (12)), True Negative Rate (TNR/specificity, Equation (13)), Positive Predictive Value (PPV/Precision, Equation (14)), Negative Predictive Value (NPV) (Equation (15)), False Positive Rate (FPR/fall-out, Equation (16)), False Negative Rate (FNR, Equation (17)) and F1-score (Equation (18)) over our dataset.

(11) $$\text{Accuracy}=\frac{\text{TP}+\text{TN}}{\text{TP}+\text{FP}+\text{FN}+\text{TN}}$$

(12) $$\text{TPR}=\frac{\text{TP}}{\text{TP}+\text{FN}}$$

(13) $$\text{TNR}=\frac{\text{TN}}{\text{FP}+\text{TN}}$$

(14) $$\text{PPV}=\frac{\text{TP}}{\text{TP}+\text{FP}}$$

(15) $$\text{NPV}=\frac{\text{FP}}{\text{FP}+\text{TN}}\;$$

(16) $$\text{FPR}=\frac{\text{FP}}{\text{FP}+\text{TN}}=1-\text{TNR}$$

(17) $$\text{FNR}=\frac{\text{FN}}{\text{TP}+\text{FN}}=1-\text{TPR}$$

(18) $$F1\text{ score}=\frac{2\text{TP}}{2\text{TP}+\text{FP}+\text{FN}}\;$$

In the equations above, TP stands for True Positive (predicted hot-spots that are actual hot-spots), FP stands for False Positive (predicted hot-spots that are not actual hot-spots), FN stands for False Negative (non-predicted hot-spots that are actual hot-spots) and TN stands the True Negatives (correctly-predicted null-spots).

### 4.4. Comparison with Other Software

We compared our results with some of the common methods in the literature: Robetta [19], KFC2-A [20] and KFC2-B [20] and CPORT [21].

## 5. Conclusions

In conclusion, we were thus able to train an accurate and robust predictor using c-forest, a random forest ensemble learning method, and up-sampling of the minor class (HS) for dataset balance. This new method can now be widely applied to the detection of HS in protein-protein interfaces. The code is available upon request, will be implemented as a web-server in the near future and made available for the scientific community at the HADDOCK GitHub repository (http:github.com/haddocking).

## Figures and Tables

**Table 1:** Statistical metrics attained for five algorithms with top performance for each of the studied conditions for the training set.

Pre-Processing	Metrics	Algorithms				
Scaled		Nnet	avNNET	C5.0 Tree	C5.0 Rules	svmRadialSigma
	AUROC	0.52	0.65	0.77	0.72	0.78
	Accuracy	0.92	0.94	0.96	0.92	0.91
	Sensitivity	0.92	0.88	0.88	0.85	0.80
	Specificity	0.91	0.98	1.00	0.96	0.97
	PPV	0.86	0.95	0.99	0.92	0.93
	NPV	0.95	0.94	0.94	0.92	0.89
	FPR	0.09	0.02	0.00	0.04	0.03
	*F*1-score	0.89	0.92	0.93	0.89	0.86
Scaled_Down		c-Forest	avNNET	C5.0Tree	C5.0Rules	GBM
	AUROC	0.79	0.70	0.73	0.71	0.80
	Accuracy	0.91	0.95	0.96	0.90	1.00
	Sensitivity	0.93	0.96	0.96	0.89	0.99
	Specificity	0.90	0.93	0.95	0.91	1.00
	PPV	0.90	0.93	0.95	0.9	1.00
	NPV	0.92	0.96	0.96	0.89	0.99
	FPR	0.1	0.07	0.05	0.09	0
	F1-score	0.91	0.95	0.96	0.9	1.00
Scaled_Up		c-Forest	avNNET	C5.0Tree	C5.0Rules	GBM
	AUROC	0.85	0.75	0.85	0.82	0.84
	Accuracy	0.93	0.94	0.98	0.95	0.98
	Sensitivity	0.93	0.96	0.99	0.96	0.97
	Specificity	0.93	0.92	0.97	0.94	0.99
	PPV	0.93	0.92	0.97	0.94	0.99
	NPV	0.93	0.96	0.99	0.95	0.97
	FPR	0.07	0.08	0.03	0.06	0.01
	F1-score	0.93	0.94	0.98	0.95	0.98
PCA		nnet	avNNET	C5.0Tree	C5.0Rules	svmRadialSigma
	AUROC	0.69	0.75	0.61	0.59	0.76
	Accuracy	1.00	0.99	0.98	0.92	0.91
	Sensitivity	1.00	0.97	0.98	0.91	0.76
	Specificity	1.00	1.00	0.98	0.93	0.99
	PPV	1.00	0.99	0.96	0.89	0.97
	NPV	1.00	0.98	0.99	0.95	0.88
	FPR	0	0	0.02	0.07	0.01
	F1-score	1.00	0.98	0.97	0.90	0.85
PCA_Down		nnet	avNNET	C5.0Tree	C5.0Rules	svmRadialSigma
	AUROC	0.70	0.78	0.67	0.67	0.75
	Accuracy	0.87	0.91	0.97	0.91	0.91
	Sensitivity	0.88	0.88	0.96	0.96	0.88
	Specificity	0.87	0.93	0.99	0.87	0.93
	PPV	0.87	0.92	0.99	0.88	0.93
	NPV	0.88	0.89	0.96	0.95	0.89
	FPR	0.13	0.07	0.01	0.13	0.07
	F1-score	0.87	0.90	0.97	0.92	0.91
PCA_Up		nnet	avNNET	C5.0Tree	C5.0Rules	svmRadialSigma
	AUROC	0.75	0.82	0.80	0.78	0.80
	Accuracy	0.95	0.98	0.98	0.96	0.94
	Sensitivity	0.94	0.97	0.99	0.96	0.92
	Specificity	0.96	0.99	0.98	0.96	0.95
	PPV	0.96	0.99	0.98	0.96	0.95
	NPV	0.94	0.97	0.99	0.96	0.92
	FPR	0.04	0.01	0.02	0.04	0.05
	F1-score	0.95	0.98	0.98	0.96	0.94

avNNET: model averaged Neural Network; C5.0 Rules (single C5.0 Ruleset); C5.0 Tree (single C5.0 Tree); c-forest (conditional inference random forest); GBM (stochastic gradient boosting machine); nnet (neuronal network); svmRadialSigma (support vector machines with the Radial basis function kernel); Positive Predictive Value (PPV); Negative Predictive Value (NPV); False Positive Rate (FPR).

**Table 2:** Statistical metrics attained for 5 algorithms with the top performance for each of the studied conditions for the independent test set.

Pre-Processing	Metrics	Algorithms				
Scaled		Nnet	avNNET	C5.0 Tree	C5.0 Rules	svmRadialSigma
	AUROC	0.71	0.68	0.68	0.72	0.70
	Accuracy	0.74	0.71	0.71	0.74	0.73
	Sensitivity	0.57	0.57	0.5	0.60	0.55
	Specificity	0.83	0.79	0.83	0.82	0.83
	PPV	0.65	0.6	0.62	0.65	0.64
	NPV	0.78	0.77	0.75	0.79	0.77
	FPR	0.43	0.43	0.4	0.4	0.45
	F1-score	0.61	0.58	0.55	0.62	0.59
Scaled_Down		c-forest	avNNET	C5.0 Tree	C5.0 Rules	GBM
	AUROC	0.75	0.68	0.63	0.71	0.73
	Accuracy	0.76	0.69	0.64	0.72	0.75
	Sensitivity	0.79	0.71	0.67	0.76	0.74
	Specificity	0.74	0.69	0.62	0.70	0.75
	PPV	0.63	0.55	0.49	0.59	0.62
	NPV	0.87	0.81	0.77	0.84	0.84
	FPR	0.21	0.29	0.33	0.24	0.26
	F1-score	0.7	0.62	0.57	0.66	0.68
Scaled_Up		c-forest	AvNNET	C5.0 Tree	C5.0 Rules	GBM
	AUROC	0.78	0.73	0.65	0.70	0.80
	Accuracy	0.80	0.75	0.69	0.73	0.82
	Sensitivity	0.76	0.66	0.48	0.59	0.76
	Specificity	0.82	0.80	0.80	0.81	0.85
	PPV	0.70	0.64	0.57	0.63	0.73
	NPV	0.86	0.81	0.74	0.78	0.86
	FPR	0.24	0.34	0.52	0.41	0.24
	F1-score	0.73	0.65	0.52	0.61	0.75
PCA		Nnet	avNNET	C5.0 Tree	C5.0 Rules	svmRadialSigma
	AUROC	0.65	0.73	0.68	0.71	0.71
	Accuracy	0.67	0.75	0.7	0.74	0.74
	Sensitivity	0.60	0.60	0.66	0.67	0.52
	Specificity	0.71	0.84	0.72	0.77	0.86
	PPV	0.54	0.67	0.57	0.62	0.67
	NPV	0.77	0.79	0.79	0.81	0.76
	FPR	0.4	0.4	0.34	0.33	0.48
	F1-score	0.57	0.64	0.61	0.64	0.58
PCA_Down		Nnet	avNNET	C5.0 Tree	C5.0 Rules	svmRadialSigma
	AUROC	0.70	0.68	0.59	0.61	0.69
	Accuracy	0.71	0.69	0.61	0.63	0.70
	Sensitivity	0.76	0.71	0.55	0.60	0.72
	Specificity	0.68	0.69	0.64	0.64	0.69
	PPV	0.56	0.55	0.46	0.48	0.56
	NPV	0.84	0.81	0.72	0.74	0.82
	FPR	0.24	0.29	0.45	0.4	0.28
	F1-score	0.65	0.62	0.50	0.53	0.63
PCA_Up		Nnet	avNNET	C5.0 Tree	C5.0 Rules	svmRadialSigma
	AUROC	0.67	0.75	0.56	0.61	0.69
	Accuracy	0.7	0.77	0.59	0.63	0.71
	Sensitivity	0.59	0.64	0.48	0.55	0.64
	Specificity	0.76	0.84	0.65	0.68	0.75
	PPV	0.58	0.69	0.43	0.48	0.59
	NPV	0.77	0.81	0.69	0.73	0.79
	FPR	0.41	0.36	0.52	0.45	0.36
	F1-score	0.58	0.66	0.46	0.52	0.61

avNNet: model averaged Neural Network; C5.0 Rules (single C5.0 Ruleset); C5.0 Tree (single C5.0 Tree); c-forest (conditional inference random forest); GBM (stochastic gradient boosting machine); nnet (neuronal network); svmRadialSigma (support vector machines with the Radial basis function kernel).

**Table 3:** Comparison of the statistical metrics attained for the best predictor in this work and some of the most common ones in the literature.

Perfomance	Algorithms											
	c-Forest/ Up-Scaling Classes		SBHD2		Robetta		KFC2-A		KFC2-B		CPORT	
	Training	Test	Training	Test	Training	Test	Training	Test	Training	Test	Training	Test
AUROC	0.85	0.78	0.74	0.69	0.62	0.62	0.72	0.66	0.60	0.67	0.54	0.54
Accuracy	0.93	0.80	0.70	0.71	0.66	0.66	0.76	0.71	0.70	0.73	0.49	0.49
Sensitivity	0.93	0.76	0.70	0.70	0.38	0.29	0.57	0.53	0.26	0.28	0.55	0.54
Specificity	0.93	0.82	0.70	0.71	0.85	0.88	0.85	0.81	0.93	0.96	0.45	0.47
PPV	0.93	0.70	0.55	0.56	0.61	0.60	0.67	0.59	0.65	0.80	0.34	0.35
NPV	0.93	0.86	0.82	0.82	0.68	0.67	0.79	0.77	0.71	0.72	0.66	0.66
F1-score	0.93	0.73	0.62	0.62	0.47	0.39	0.62	0.56	0.37	0.42	0.42	0.42

## Supplementary Materials

Supplementary materials can be found at http://www.mdpi.com/1422-0067/17/8/1215/s1.
