# Supplementary material for: A Machine Learning Approach for Hot-Spot Detection at Protein-Protein Interfaces
Source: Int J Mol Sci. 2016 Jul 27;17(8):1215. doi: 10.3390/ijms17081215 (PMC5000613; doi:10.3390/ijms17081215)
Supplement: Supplementary file 1 [file ijms-17-01215-s001.zip › ijms-135111-Supplementary Materials/ijms-135111 SI.pdf]

Supplementary Mateirlas: A Machine Learning Approach for Hot-Spot Detection at Protein-Protein Interfaces

Rita Melo, Robert Fieldhouse, André Melo, João D. G. Correia, Maria Natália N. D. S. Cordeiro, Zeynep H. Gümüş, Joaquim Costa, Alexandre M. J. J. Bonvin and Irina S. Moreira

Table S1. $\Delta\Delta G_{binding}$ experimental values/HotSpot(HS)-NullSpots(NS) classification for the residues at our dataset.

Complex	CPX_PDBID	Reference	Mutation		
			Residue	Chain	ΔΔ*G*
Ribonuclease Inhibitor/Angiogenin	1A4Y	[47]	TRP	261	0.10
			TRP	263	1.20
			SER	289	0.00
			TRP	318	1.50
			LYS	320	−0.30
			GLU	344	0.20
			TRP	375	1.00
			GLU	401	0.90
			TYR	434	3.30
			ASP	435	3.50
			TYR	437	0.80
			ARG	457	−0.20
			ILE	459	0.70
			ARG	5	2.30
			HIS	8	0.90
			GLN	12	0.30
			HIS	13	−0.30
			ARG	31	0.20
			ARG	32	0.90
			ASN	68	0.20
			HIS	84	0.20
			TRP	89	0.20
			GLU	108	−0.30
			HIS	114	0.65
Tissue Factor/Fab(5G9)	1AHW	[48]	TYR	156	4.00
			THR	167	0.00
			THR	170	1.00
			LEU	176	1.00
			ASP	178	−0.50
			THR	197	1.30
			VAL	198	−0.30

Table S1. *Cont.*

Complex	CPX_PDBID	Reference	Mutation		
			Residue	Chain	ΔΔ*G*
Barnase/barnstar	1BRS	[49]	LYS	27	5.40
			ARG	59	5.20
			GLU	60	−0.20
			GLU	73	2.80
			ARG	87	5.50
			HIS	102	6.00
			TYR	29	3.40
			ASP	35	4.50
			ASP	39	7.70
			THR	42	1.80
			GLU	76	1.30
E. coli colicin E9 dnase domain/ cognate immunity protein IM9	1BXI	[50]	CYS	23	0.92
			ASN	24	0.14
			THR	27	0.73
			SER	28	0.17
			SER	29	0.96
			GLU	30	1.14
			LEU	33	3.42
			VAL	34	2.58
			VAL	37	1.66
			THR	38	0.90
			GLU	41	2.08
			SER	48	0.01
			GLY	49	1.49
			SER	50	2.19
			ASP	51	5.92
			TYR	55	4.63
			PRO	56	1.24
Bovine α-chymotrypsin/BPTI	1CBW	[51]	THR	11	0.20
			LYS	15	2.00
			ARG	17	0.50
			ILE	19	0.10
			VAL	34	0.00
			ARG	39	0.20

Table S1. *Cont.*

Complex	CPX_PDBID	Reference	Mutation		
			Residue	Chain	ΔΔ*G*
Factor VIIA/Tissue factor	1DAN	[52]	LYS	15	−0.40
			THR	17	0.10
			ASN	18	0.20
			LYS	20	2.60
			THR	21	−0.20
			ILE	22	0.70
			GLU	24	0.70
			LYS	41	−0.04
			SER	42	−0.05
			ASP	44	0.70
			LYS	46	0.25
			SER	47	0.05
			LYS	48	0.40
			PHE	50	0.40
			ASP	58	2.18
			LYS	68	−0.10
IgG1-κ D1.3 Fv/E5.2 Fv	1DVF	[53]	HIS	30	1.70
			TYR	32	2.00
			TYR	49	1.70
			TYR	50	0.70
			TRP	92	0.30
			SER	93	1.20
			THR	30	0.90
			TYR	32	1.80
			TRP	52	4.20
			ASP	54	4.30
			ASN	56	1.20
			ASP	58	1.60
			GLU	98	4.20
			ARG	99	1.90
α-Thrombin/thrombomodulin	1DX5	[54]	ILE	24	NS
			LYS	235	NS
			PHE	34	2.60
			LYS	36	NS
			PRO	37	NS
			GLN	38	NS
			GLU	39	NS
			LEU	65	NS
			ARG	67	3.4
			THR	74	NS
			ARG	75	NS
			TYR	76	3.00
			GLU	80	HS
			LYS	81	NS
			ILE	82	2.6
			MET	84	0.3
			LYS	110	0.00

Table S1. *Cont.*

Complex	CPX_PDBID	Reference	Mutation		
			Residue	Chain	ΔΔG
HIV gp120/CD4	1GC1	[55]	SER	23	0.29
			GLN	25	0.03
			HIS	27	0.28
			LYS	29	0.59
			ASN	32	0.18
			GLN	33	0.10
			LYS	35	0.32
			GLN	40	−0.41
			SER	42	0.00
			LEU	44	1.04
			THR	45	−0.15
			ASN	52	0.70
			ARG	59	1.16
			SER	60	−0.09
			ASP	63	−0.32
			GLN	64	0.44
			GLU	85	1.31
Subtype N9 neuraminidase/ Antibody NC10	1NMB	[56]	ASP	56	2.80
			TYR	99	2.13
			THR	93	0.30
IgG1-κ D1.3 Fv/HEW lysozyme	1VFB	[57]	HIS	30	0.80
			TYR	32	1.30
			TYR	49	0.80
			TYR	50	0.40
			THR	53	−0.23
			TRP	92	2.70
			SER	93	0.30
			THR	30	0.10
			TYR	32	0.50
			TRP	52	0.40
			ARG	99	0.10
			ASP	100	3.10
			TYR	101	4.00
			ASP	18	0.30
			ASN	19	0.30
			TYR	23	0.40
			SER	24	0.80
			LYS	116	0.70
			THR	118	0.80
			ASP	119	1.00
			VAL	120	0.90
			GLN	121	2.90
			ILE	124	1.20
			ARG	125	1.80
			LEU	129	0.20

Table S1. *Cont.*

Complex	CPX_PDBID	Reference	Mutation		
			Residue	Chain	ΔΔG
HyHEL-10/HEW Lysozyme	3HFM	[58]	SER	31	0.20
			ASP	32	2.00
			TYR	33	6.00
			TYR	50	7.50
			TYR	53	3.29
			TYR	58	1.70
			TYR	20	5.00
			ARG	21	1.00
			TRP	63	0.30
			ARG	73	−0.20
			LEU	75	1.25
			THR	89	0.00
			ASN	93	0.60
			LYS	96	7.00
			LYS	97	6.00
			SER	100	0.25
			ASP	101	1.02
			HIS	15	-0.50
			ASN	31	5.25
			ASN	32	5.20
			TYR	50	4.60
			GLN	53	1.00
			TYR	96	2.80
Protein A/Z/IgG1 MO61 Fc	1FC2	[59]	ASN	147	0.60
			ILE	150	2.20
			LYS	154	1.20
Ribonuclease A/Ribonuclease inhibitor	1DFJ	[60]	GLU	202	1.00
			TRP	257	1.30
			TRP	259	2.20
			GLU	283	1.30
			SER	285	0.80
			TRP	314	1.00
			LYS	316	1.30
			GLU	340	1.60
			GLU	397	1.30
			TYR	430	5.90
			ASP	431	3.60
			TYR	433	2.60
			ARG	453	0.80
			GLU	202	1.00
			TRP	257	1.30

Table S1. *Cont.*

Complex	CPX_PDBID	Reference	Mutation		
			**Residue**	**Chain**	**ΔΔ*G***
Integrin α2 I domain/collagen	1DZI	[61]	ASN	154	NS
			TYR	157	NS
			GLN	215	HS
			ASP	219	NS
			LEU	220	NS
			THR	221	HS
			GLU	256	NS
			HIS	258	NS
BMP-2/BMP receptor IA extracellular domains	1ES7	[62]	PHE	49	NS
			PRO	50	NS
			VAL	26	NS
			TRP	31	HS
NIDOGEN-1/PERLECAN IG3	1GL4	[63]	ARG	403	NS
			ASP	427	HS
			HIS	429	HS
			TYR	431	HS
			TYR	440	NS
			GLU	616	HS
			ARG	620	HS
MazE (antidote)/MazF (toxin)	1UB4	[64]	PHE	453	NS
			LEU	455	HS
			LEU	458	HS
IGG1 FC/streptococcal protein G	1FCC	[65]	THR	25	0.24
			GLU	27	>4.90
			LYS	28	1.30
			LYS	31	3.50
			ASN	35	NS
			ASP	40	0.30
			GLU	42	0.40
			TRP	43	3.80
Oligomerization domain of P53	3SAK	[66]	GLU	8	NS
			PHE	10	HS
			THR	11	NS
			LEU	12	HS
			GLN	13	NS
			ILE	14	HS
			ARG	15	NS
			ARG	17	NS
			PHE	20	HS
			PHE	23	HS
			LEU	26	HS
			ASN	27	NS
			LEU	30	HS
			ASP	34	NS

Table S1. *Cont.*

Complex	CPX_PDBID	Reference	Mutation		
			Residue	Chain	ΔΔ*G*
Factor VIIA/Tissue factor	1FAK	[67]	ASN	37	NS
			LYS	41	NS
			SER	42	NS
			ASP	44	NS
			TYR	94	NS
			LYS	15	−0.40
			THR	17	0.10
			ASN	18	0.20
			LYS	20	2.60
			ILE	22	1.70
			GLU	24	NS
			SER	47	0.10
			LYS	48	0.40
			PHE	50	0.40
			ASP	58	2.50
			GLU	128	0.10
			LEU	133	0.10
			ARG	135	0.50
			PHE	140	1.30
			THR	203	0.10
			VAL	207	NS
Subtilisin BPN' precursor/ chymotrypsin inhibitor 2	1TM1	[68]	THR	58	2.64
			MET	59	1.02
			GLU	60	2.98
			TYR	61	2.57
			ARG	62	1.25
			ARG	65	3.40
			ARG	67	2.99
			VAL	70	0.02
Interleukin-4/Interleukin-4 receptor α chain	1IAR	[69]	ILE	5	0.22
			THR	6	1.17
			GLN	8	−0.10
			ILE	11	−0.22
			THR	13	0.07
			ASN	15	0.97
			SER	16	−0.03
			GLU	19	−0.18
			LYS	77	−0.32
			GLN	78	0.15
			ARG	81	0.12
			PHR	82	0.48
			LYS	84	−0.90
			ARG	85	0.34
			ARG	88	0.42
			ASN	89	3.74
			TRP	91	1.55

Table S1. *Cont.*

Complex	CPX_PDBID	Reference	Mutation		
			Residue	Chain	ΔΔ*G*
14.3.D T cell antigen receptor/ Staphylococcal enterotoxin C3	1JCK	[70]	THR	20	1.65
			TYR	26	1.77
			ASN	60	1.64
			TYR	90	2.89
			VAL	91	2.22
			LYS	103	0.67
			PHE	176	2.13
Growth factor receptor-bound protein 2/Vav proto-oncogene	1GCQ	[71]	PRO	595	0.76
			PRO	608	1.31
			PRO	609	0.12
			PRO	657	0.08
Cyclophilin A/HIV-1 capsid	1AK4	[72]	PRO	485	2.44
			VAL	486	2.35
			HIS	487	2.36
			GLY	489	3.43
			PRO	490	3.52
			ILE	491	1.60
			PRO	493	2.04
ATF-urokinase receptor	2I9B	[73]	ARG	137	−0.29
			LYS	139	0.67
			ARG	142	0.36
			HIS	143	0.66
			ARG	145	0.41
Lyzozyme C/inhibitor	1UUZ	[74]	CYS	64	0.65
Mlc/ EIICB	3BP8	[75]	PHE	136	0.71
IMME2/ E9 DNASE	2WPT	[76]	GLU	30	1.73
			VAL	37	3.79
			GLU	41	4.48
			SER	50	2.42
			PRO	56	2.92
			ARG	54	0.87
			ASN	72	0.70
			SER	74	−0.13
			ASN	75	1.25
			SER	77	−0.46
			SER	78	−0.09
			SER	84	−0.07
			PHE	86	1.05
			THR	87	0.38
			GLN	92	0.38
			LYS	97	0.65
			VAL	98	0.26
Cytochrome C peroxidase/ Cytochrome C	2PCC	[77]	ASP	34	−0.89
			VAL	197	2.09
			GLU	290	6.18
			LYS	87	0.90

Table S1. *Cont.*

Complex	CPX_PDBID	Reference	Mutation		
			Residue	Chain	ΔΔ*G*
JEL42 FAB/HPR	2JEL	[78]	THR	62	0.00
			GLU	68	0.41
			GLU	70	2.72
			HIS	76	−0.41
			GLU	83	0.00
Nuclease A/inhibitor	2O3B	[79]	GLU	24	5.45
			GLN	74	3.22
			TRP	76	4.06
Profilin/β-Actin	2BTF	[80]	PHE	59	4.27
			LYS	125	0.00
UCHL3/UbVME	1XD3	[81]	LYS	6	1.64
			LEU	8	2.10
			GLU	24	1.59
			LYS	27	0.46
			ASP	39	1.34
			ILE	44	2.47
			GLU	51	−0.24
			ASP	52	−0.06
			ASP	58	−0.41
TSG101(UEV)/ubiquitin	1S1Q	[82]	VAL	43	0.67
			PHE	44	0.20
			ASN	45	1.23
			ASP	46	0.96
			TRP	75	0.27
			PHE	88	0.77
RALGDS/RAS	1LFD	[83]	ARG	20	1.13
			LYS	32	1.32
			LYS	48	0.26
			ASP	51	−0.58
			LYS	52	1.17
			ASP	56	−0.28
			GLU	57	−0.25

Table S1. *Cont.*

Complex	CPX_PDBID	Reference	Mutation		
			Residue	Chain	ΔΔ*G*
TGF-BETA3/ TBR-2	1KTZ	[84]	ARG	25	1.48
			ARG	94	2.87
			LEU	27	2.26
			PHE	30	3.41
			ASP	32	1.96
			ASN	47	0.72
			SER	49	0.78
			ILE	50	2.33
			THR	51	1.95
			SER	52	0.66
			ILE	53	1.81
			GLU	55	1.66
			VAL	62	1.09
			GLU	75	1.52
			VAL	77	0.86
			HIS	79	0.74
			PHE	110	1.37
			MET	112	1.31
			ASP	118	1.26
			GLU	119	1.93
			ILE	125	0.98
AML1/CBF-BETA	1H9D	[85]	ARG	3	1.16
			VAL	4	1.40
			GLY	61	2.07
			GLN	67	1.36
			LEU	103	0.94
			ASN	104	2.29
Chemotaxis protein Chey/Chea	1FFW	[86]	GLU	171	0.71
			GLU	178	0.64
			HIS	181	0.03
			ASP	202	−0.07
			ASP	207	0.10
			CYS	213	0.20
			PHE	214	3.63
			ILE	216	0.43

Table S1. *Cont.*

Complex	CPX_PDBID	Reference	Mutation		
			Residue	Chain	ΔΔ*G*
MT-SP1/ S4 FAB	3NPS	[87]	GLN	38	0.03
			ILE	41	0.64
			ARG	87	−0.15
			PHE	94	1.59
			ASN	95	0.25
			ASP	96	1.50
			PHE	97	0.46
			THR	98	0.72
			HIS	143	1.87
			GLN	145	0.29
			TYR	146	1.77
			THR	150	0.17
			GLU	169	0.61
			GLN	177	−0.06
			GLN	175	0.74
			ASP	217	1.46
			ARG	222	−0.08
			LYS	224	−0.10
β-Trypsin/BPTI	2FTL	[88]	GLY	12	4.37
			LYS	15	10.36
			ILE	18	5.00
			GLY	36	2.01
RNASE 1/RNASE inhibitor	1Z7X	[89]	GLU	206	1.01
			TRP	261	1.33
			TRP	263	2.20
			GLU	287	1.32
			SER	289	0.81
			TRP	318	0.99
			LYS	320	1.32
			GLU	344	1.56
			TRP	375	1.66
			GLU	401	1.30
			TYR	434	5.93
			ASP	435	3.65
			TYR	437	2.61
			ARG	457	0.84
			ILE	459	0.34
Human leukocyte elastase/OMTKY3	1PPF	[90]	LYS	13	0.75
			PRO	14	−0.12
			THR	17	3.18
			LEU	18	1.01
			GLU	19	1.20
			TYR	20	3.20
			ARG	21	0.21
			GLY	32	0.26
			ASN	36	−1.64

Table S1. *Cont.*

Complex	CPX_PDBID	Reference	Mutation		
			**Residue**	**Chain**	**ΔΔG**
Proteinase B/OMTKY3	3SGB	[91]	LYS	13	−2.56
			PRO	14	−0.19
			THR	17	3.40
			LEU	18	2.96
			GLU	19	1.02
			TYR	20	1.94
			ARG	21	0.05
			GLY	32	1.29
			ASN	36	0.33
Efb-C/C3d	2GOX	[92]	ARG	131	2.25
			ASN	138	1.57
Interstitial collagenase/ Metalloproteinase inhibitor 1	2J0T	[93]	VAL	4	0.00
			SER	68	2.11
			THR	2	4.29
			MET	66	1.64
Bone morphogenetic protein 2/ Crossveinless 2	3BK3	[94]	LEU	1	0.00
			ILE	2	1.04
			ILE	18	0.49
			ILE	21	1.31
			ILE	27	1.26
Membrane-type serine protease 1/BPTI	1EAW	[95]	GLN	38	−0.52
			ILE	41	−0.82
			ILE	60	−0.19
			ASP	60A	−0.17
			ASP	60B	1.50
			ARG	60C	0.59
			PHE	60E	−0.43
			ARG	60F	0.23
			TYR	60G	−0.08
			ARG	87	−0.15
			PHE	94	0.73
			ASN	95	0.31
			ASP	96	0.65
			PHE	97	0.89
			THR	98	0.25
			HIS	143	−0.01
			GLN	145	0.31
			TYR	146	0.50
			THR	150	0.09
			LEU	153	0.50
			GLU	169	0.70
			GLN	174	0.56
			GLN	175	−0.13
			ASP	217	2.23
			GLN	221A	0.14
			ARG	222	−0.09
			LYS	224	0.48

Table S1. *Cont.*

Complex	CPX_PDBID	Reference	Mutation		
			Residue	Chain	ΔΔ*G*
Membrane-type serine protease 1/E2 Fab	3BN9	[96]	GLN	38	−0.42
			ILE	41	0.00
			ILE	60	0.84
			ASP	60a	0.42
			ASP	60b	0.31
			ARG	60c	−0.04
			PHE	60e	−0.04
			ARG	60f	−0.07
			TYR	60g	0.02
			ARG	87	−0.16
			PHE	94	0.64
			ASN	95	0.77
			THR	98	1.13
			HIS	143	0.09
			GLN	145	0.13
			TYR	146	1.08
			THR	150	0.29
			LEU	153	0.34
			GLU	169	0.37
			GLN	174	−0.03
			GLN	175	2.51
			ASP	217	0.57
			GLN	221a	0.71
			ARG	222	−0.09
			LYS	224	0.78
HyHEL-63 Fab/HEW Lysozyme	1DQJ	[97]	TYR	20	3.29
			ARG	21	1.21
			LYS	97	3.52
			ASP	101	1.45
			TRP	62	0.76
			TRP	63	1.35
			LEU	75	1.45
			THR	89	0.84
			ASN	93	0.65
			LYS	96	6.16
			LYS	97	3.52
			SER	100	0.78
			ASP	101	1.30
			ASN	31	2.01
			ASN	32	4.09
			TYR	50	2.68
			SER	91	1.43
			TYR	96	1.14
			ASP	32	2.01
			TYR	33	5.52
			TYR	50	6.89
			TYR	53	1.18
			TRP	98	4.93

Table S1. *Cont.*

Complex	CPX_PDBID	Reference	Mutation		
			**Residue**	**Chain**	**ΔΔ*G***
SHV-1 β-lactamase/BLIP	2G2U	[98]	GLU	31	0.65
			SER	35	−0.95
			PHE	36	2.76
			SER	39	−0.96
			HIS	41	1.72
			GLY	48	−0.43
			TYR	50	−2.07
			TYR	51	−0.63
			TYR	53	2.30
			SER	71	−0.51
			GLU	73	−1.98
			LYS	74	−0.22
			TRP	112	0.96
			SER	113	−0.61
			GLY	141	−0.41
			PHE	142	0.28
			TYR	143	−1.85
			ARG	144	−0.34
			HIS	148	1.12
			TRP	150	1.78
			ARG	160	0.67
			TRP	162	0.53
			SER	12	1.90
			THR	10	2.05
			ILE	13	3.51
Bovine α-chymotrypsin/ Turkey ovomucoid third domain	1CHO	[99]	GLY	32	−0.77
			THR	17	4.32
			LEU	18	4.93
